# Emergence of a Novel CRESS‐DNA Virus Associated with Swine Reproductive Failure in China

**DOI:** 10.1155/tbed/4053892

**Published:** 2026-02-06

**Authors:** Xianhui Liu, Yixuan Li, Jian Xiao, Xinming Zhang, Yilong Liu, Zan li, Lin Wang, Leyi Zhang, Yanling Liu, Pengshuai Liang, Zheng Xu, Yebing Liu, Changxu Song

**Affiliations:** ^1^ State Key Laboratory of Swine and Poultry Breeding Industry, National Engineering Research Center for Breeding Swine Industry, College of Animal Science, South China Agricultural University, Guangzhou, China, scau.edu.cn; ^2^ Division of Viral Biologics Testing, China Institute of Veterinary Drug Control, Beijing, China; ^3^ State Key Laboratory of Virology and National Virus Resource Center, Wuhan Institute of Virology, Chinese Academy of Sciences, Wuhan, China, cas.cn

**Keywords:** CRESS DNA viruses, megalocircovirus, swine reproductive failure

## Abstract

The continuous emergence of circular Rep‐encoding single‐stranded (CRESS) DNA viruses across diverse hosts has been closely associated with the occurrence of severe diseases. Four circoviruses within the genus Circovirus have been identified in pigs, including porcine circovirus Type 1 (PCV1), PCV2, PCV3, PCV4, and PCV5. In late 2021, a large pig farm experienced an outbreak of reproductive disorders that were undiagnosed by standard tests. Subsequent viral metagenomic analysis of stillborn piglets identified a novel single‐stranded circular DNA virus, designated porcine megalocircovirus (PMCV). PMCV has a large genome of 9426 nt and encodes nine open reading frames. Biochemical analyses of Rep confirm PMCV as a CRESS DNA virus. However, PMCV Rep showed low amino acid sequence identities to the four PCV species and several human CRESS DNA viruses, with the highest identity of 23.6% to PCV4 Rep. The genetic evolutionary tree indicates that PMCV belongs to an unknown family of the CRESS DNA viruses. The positive detection rate for PMCV in tested samples was 24% (30/125), while the positive rate regarding pig farms was 41.18% (14/34) in China. The emergence of PMCV warrants further investigation.

## 1. Introduction

Eukaryotic circular Rep‐encoding single‐stranded (CRESS) DNA viruses encoded by eukaryotic replication proteins (Rep) are characterized by small genomes, high prevalence, and a wide range of infected hosts [1]. The term CRESS DNA virus started to appear in references since 2012 [2]. According to the International Committee on Taxonomy of Viruses (ICTV), CRESS DNA viruses have seven family members: *Circoviridae*, *Nanoviridae*, *Smacoviridae*, *Genomoviridae*, *Bacilladnaviridae*, and *Geminiviridae* [2–4].

A novel sequence represents a new species when the percentage of nucleotides is below the species threshold when compared to the entire genome of any identified species. Rep is the only gene with homology in CRESS DNA viruses and is therefore widely used in phylogenetic analyses and virus classifications [4, 5]. In genetic evolutionary analyses, a Rep protein greater than 0.20 classifies a new virus [2, 6]. The Rep protein of CRESS DNA viruses identified by viral metagenomic sequencing contains two unique functional structural domains: the HUH (his‐hydrophobic‐his) nucleic acid endonuclease domain at the N‐terminus and the Superfamily 3 (SF3) deconjugate enzyme structural domain at the C‐terminus [7]. The HUH endonuclease structural domain is characterized by the rolling circle replication (RCR) motifs I to III, which are important for the RCR initiation and termination. The SF3 deconjugase structural domain contains Walker A, Walker B, and motif C that may allow Rep to act as a replicative deconjugase during the RCR process [4].

The widely studied family of CRESS DNA viruses, the *Circoviridae*, comprises a total of two genera: the Cyclovirus genera and Circovirus genera. The family Circoviridae comprises highly diverse viral species and has an extensive host range, covering various vertebrates such as pigs, dogs, and poultry, as well as insects [8, 9]. Circoviruses mainly encode Cap and Rep. Cap is the only protein component of the viral capsid and acts as a structural protein to form the viral capsid, which is capable of self‐assembling to form icosahedral virus‐like particles (VLPs) [10]. Cap plays a crucial role in the entire viral replication cycle. Cap is involved in viral attachment, cell entry, genome packaging, and the assembly of mature viral particles. Rep mediates the recognition of viral genome replication initiation sites and utilizes its nucleic acid endonuclease activity to break circular DNA for roll‐over replication [2]. These known CRESS DNA viruses identified in pigs include porcine circovirus Type 1 (PCV1) [11], PCV2 [11], PCV3 [12], PCV4 [13], Porcine Circovirus type 5 (PCV5)[14], and Porcine Circovirus‐like virus (PCLV) [15, 16].

PCV1 is nonpathogenic and widely prevalent in swine populations. PCV2 was first identified in 1998 and is the primary pathogen responsible for porcine circovirus associated diseases (PCVDs). PCV2 can cause postweaning multisystemic wasting syndrome (PMWS), respiratory disease syndrome, reproductive disorders, and porcine dermatitis and nephropathy syndrome (PDNS) in weaned piglets [17]. PCV3 was first identified in 2016 and has been primarily associated with PDNS and reproductive disorders [18]. Subsequently, PCV4 was reported in 2019 and linked to clinical manifestations including respiratory disease, diarrhea, and PDNS [13]. Meanwhile, a novel porcine CRESS DNA virus, PCLV, has been associated with porcine diarrhea and hemorrhagic enteritis [19]. PCV5 is a recently identified novel CRESS‐DNA virus. Although its genomic structure resembles that of circoviruses, phylogenetic analysis reveals that it does not belong to the *Circoviridae* family but rather constitutes an independent branch. Epidemiological and clinical association studies demonstrate that PCV5 is widespread in swine populations in south China and is closely linked to clinical symptoms of porcine circovirus‐associated disease (PCVAD), including respiratory, diarrhea, and reproductive disorders, highlighting its significance as an emerging pathogenic virus [14]. The ability of circoviruses is to utilize genomes of limited base length to replicate in a wide range of eukaryotes and cause many important diseases. Circovirus is an excellent research model arising from the natural evolution of viruses and the molecular mechanisms by which the virus replicates and the immune mechanisms of the infected host are worthy of in‐depth investigation.

Advances in virus research and metagenomics have led to the emergence of the concepts of “viral metagenomic sequencing” [20]. For viral metagenomics analysis, VLPs are enriched from samples. The gene sequence information of the samples is obtained by high throughput sequencing technology. Various analytical methods of bioinformatics are utilized to analyze the abundance, diversity, functional analysis, and host prediction of viruses, so that we can understand the characteristics of the viral group and the roles they play in the microbiota [21, 22]. Numerous unclassified CRESS DNA viruses have been identified using viral metagenomic sequencing approaches [6, 16, 23, 24]. The number of eukaryotic CRESS DNA viruses in GenBank has more than doubled in the last 10 years [7]. It also illustrates that CRESS DNA viruses differ markedly in genomic characterization, evolution, and pathogenicity [25]. It is important to utilize viral metagenomic sequencing to understand the origin and evolutionary patterns, genetic diversity, and geographic distribution of CRESS viruses.

In this study, we identified a novel CRESS DNA virus, designated porcine megalocircovirus (PMCV), which is associated with reproductive disorders diseases. We provide a preliminary characterization of the genomes of these viruses and their prevalence in southern China.

## 2. Materials and Methods

### 2.1. Sample Processing and Nucleic Acid Extraction

Outbreaks of reproductive disorders in a large‐scale pig farm of about 3000 sows in the Guangdong Province, with no clear etiology identified in aborted stillbirths (Supporting Information Figure S1). The collected stillborn fetuses were thawed at 4°C, and approximately 100 mg of tissue samples were added to a 500 mL PBS EP tube containing 1% penicillin and 1% streptomycin. The samples were thoroughly ground on ice with a grinding rod, and the ground samples were placed in a centrifuge tube and repeatedly frozen and thawed at −80°C and 4°C for three times, and then centrifuged at 4°C, 16,100 g, for 15 min. Samples are filtered using 0.22 µm membranes. Two hundred microliters of the sample treated in the previous step was aspirated, and the viral nucleic acids were extracted using a viral nucleic acid extraction kit (Magen). The extracted viral nucleic acids were stored at −80°C for subsequent experiments.

### 2.2. Viral Metagenomic Sequencing

For viral metagenomic analysis, total RNA was extracted from the porcine stillborn fetuses. Extracted RNA purity and concentration were measured by Nanodrop (OD260/280) to assess the integrity of the RNA in the extracted samples. Ribosomal RNA (rRNA) was removed from the samples using a kit to remove host sequences. The rRNA depleted RNA was randomly interrupted and used as a template to synthesize the first strand cDNA with a six‐base free primer. Complementary strand cDNA was synthesized by adding buffer, dNTPs, and DNA polymerase I and RNase H. The double‐stranded cDNA was purified with AMPure XP beads. The purified double‐stranded cDNA is first end‐repaired, A‐base added and connected to the sequencing junction, and then fragment‐selected with AMPure XP magnetic beads. Finally, the U‐containing strand was degraded and the cDNA library was enriched by PCR. After library construction was completed, preliminary quantification was first performed using Qubit 2.0 to dilute the library to 2 ng/uL. Size testing of libraries was inserted using the Agilent 2100. After the insert fragment size was as expected, the effective concentration of the library was accurately quantified using a quantitative PCR method (effective concentration of the library >2 nM). The different libraries were mixed according to the target downstream data volume and sequenced using the Illumina HiSeqX10 platform with 150 nt read lengths. Raw sequencing reads were processed with adapter trimming and quality filtering using FASTP. These include reads in which the percentage of N removed is greater than 10%; reads in which the number of bases with a quality value of *Q* ≤ 10 is removed for more than 50% of the entire read. The clean reads obtained from the sequenced samples were assembled separately using CLC Genomics Workbench 11.0 into counting. Finally, Blastn and Blastx searches were used to detect possible viruses in the samples.

### 2.3. Assembly of the Complete Viral Genome

The PCR product was sequenced after linkage to a T vector, and blast analysis was performed. Based on the sequenced sequences, reverse complementary primers were designed to amplify the remaining viral genome by PCR. Sequence fragments of approximately 500 nt were obtained by viral metagenomic sequencing. Primers were designed to validate the sequence from viral metagenomic sequencing, and reverse complementary primers were designed based on the sequenced sequence, and the remaining viral genome (8800 bp) was subsequently amplified.

### 2.4. Analysis of Evolutionary Tree and Amino Acid Sequence Comparison

The reference sequence of the evolutionary tree used was downloaded from NCBI. Genome assembly used Lasergene11.1 and Snapgene7.1.2 software. Using the ClustalW calibration method, all sequences were further collated with MegAlign (Lasergene). Phylogenetic trees were built in MEGA7 software using the great likelihood method with 1000 bootstrap replications. The coding regions of the Rep proteins were compared and analyzed using the neighbor‐joining method for correction and complete gap deletion, and the maximum likelihood method for constructing an evolutionary tree. The amino acid sequences were compared using DNAman and SDTv1.2 software.

### 2.5. Expression of Rep in *Escherichia coli*

The full‐length Rep gene of PMCV was cloned into a pET28a vector. A truncated construct (Rep‐ΔN145), lacking the N‐terminal 145 amino acids, was cloned into a pGEX‐GST vector to produce an N‐terminal GST fusion protein (Table 1). Each recombinant plasmid was verified and transformed into *E. coli* BL21(DE3) cells for heterologous expression.

**Table 1:** The list of primers for plasmids construction.

Primer name	Primer sequence (5′ → 3′)
pGEXT‐ PMCV‐RepΔ145‐F	TATTTTCAGGGATCCGGTAACAAAAACGGCCGTGAAA
pGEXT‐ PMCV‐RepΔ145‐R	ATGCGGCCGCTCGAGTTAGCCCATGTTTTCGTGTTGA
pGEX‐GST‐F	GGATCCCTGAAAATACAGGTTTTCATC
pGEX‐GST‐R	CTCGAGCGGCCGCAT
pET28a‐F	GGATCCCTGAAAATACAGGTTTTCG
pET28a‐R	GTCGACAAGCTTGCGGC
PMCV REP‐F	CTGTATTTTCAGGGATCCATGAGTGAAGTTCAGCCGT
PMCV REP‐R	GCAAGCTTGTCGACTTAGCCCATGTTTTCGTGCTGA

A single positive transformant was used to inoculate a 5 mL LB starter culture, which was grown overnight at 37°C with shaking. This culture was then scaled up to 1 L of fresh, antibiotic‐containing LB medium. Cells were grown at 37°C until the OD_600_ reached 0.6–0.8. Protein expression was induced with 0.2 mM IPTG, and the temperature was shifted to 16°C for continued shaking over 16–18 h.

Cells were harvested by centrifugation (5,000 × *g*, 10 min, 4°C). The pellet was resuspended in lysis buffer (20 mM Tris‐HCl, pH 8.0, 300 mM NaCl, 5.6 mM β‐mercaptoethanol) and lysed by high‐pressure homogenization. The lysate was clarified by centrifugation (12,000 × *g*, 60 min, 4°C).

The soluble supernatant was subjected to affinity chromatography: Ni‐NTA for the GST‐tagged Rep‐ΔN145. Further purification involving ion‐exchange and size‐exclusion chromatography yielded the target protein in a highly pure and homogeneously oligomeric state.

### 2.6. ATPase Activity of Viral Rep

ATPase activity was measured using the ATPase/GTPase Activity Assay Kit (Sigma–Aldrich). A preliminary experiment was conducted to determine the optimal enzyme‐to‐substrate ratio. The reaction mixture (40 μL total volume) contained 20 μL of Assay Buffer, 10 μL of 4 mM ATP, and 10 μL of the test protein (final concentrations: 0.05, 0.1, 0.2, 0.5, and 1 μM). Controls included protein storage buffer (20 mM Tris, 100 mM NaCl) and an enzyme‐free assay buffer control. After a 30 min incubation at 37°C, 200 μL of Malachite Green reagent was added to each well, followed by an additional 30 min incubation at 37°C. Absorbance was measured at 620 nm using a microplate reader. The condition showing the highest increasing trend in activity was selected to ensure the final enzyme‐to‐substrate ratio used in the formal assay was not lower than this value.

Based on the preexperimental results, the formal assay was performed with a protein concentration of 0.5 μM and an 8‐point ATP dilution series (0, 62.5, 125, 250, 500, 1000, 2000, and 4000 μM). Each reaction (40 μL total volume) consisted of 20 μL of Assay Buffer, 10 μL of ATP (at the specified concentration), and 10 μL of the target protein (0.5 μM). All conditions were tested in triplicate. The reaction was initiated by the addition of the protein to ensure consistent timing. After 30 min of incubation at 37°C, 200 μL of Malachite Green reagent was added, and the plate was incubated for another 30 min at 37°C before measuring the absorbance at 620 nm.

## 3. Results

### 3.1. Viral Metagenomic Sequencing

**Figure 1:**
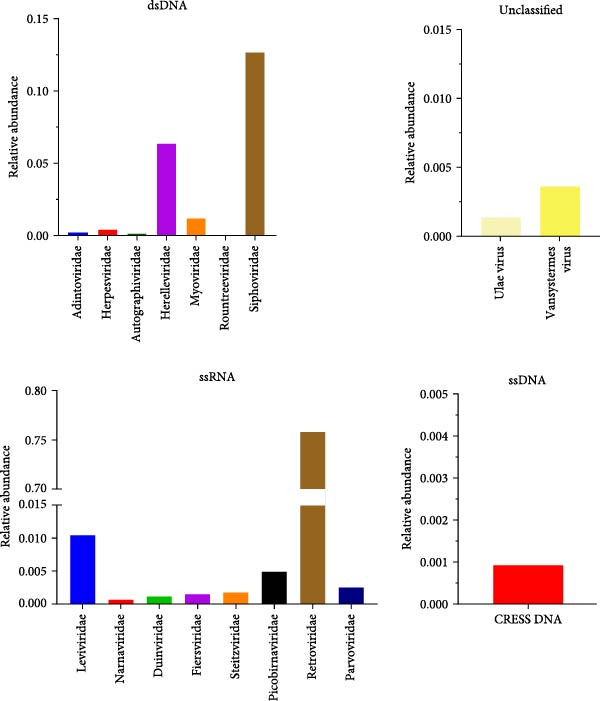
Viral communities of stillborn pigs with reproductive disorders and the viruses belonging to different families: DNA and RNA viruses of identified families and unassigned viruses.

**Figure 2:**
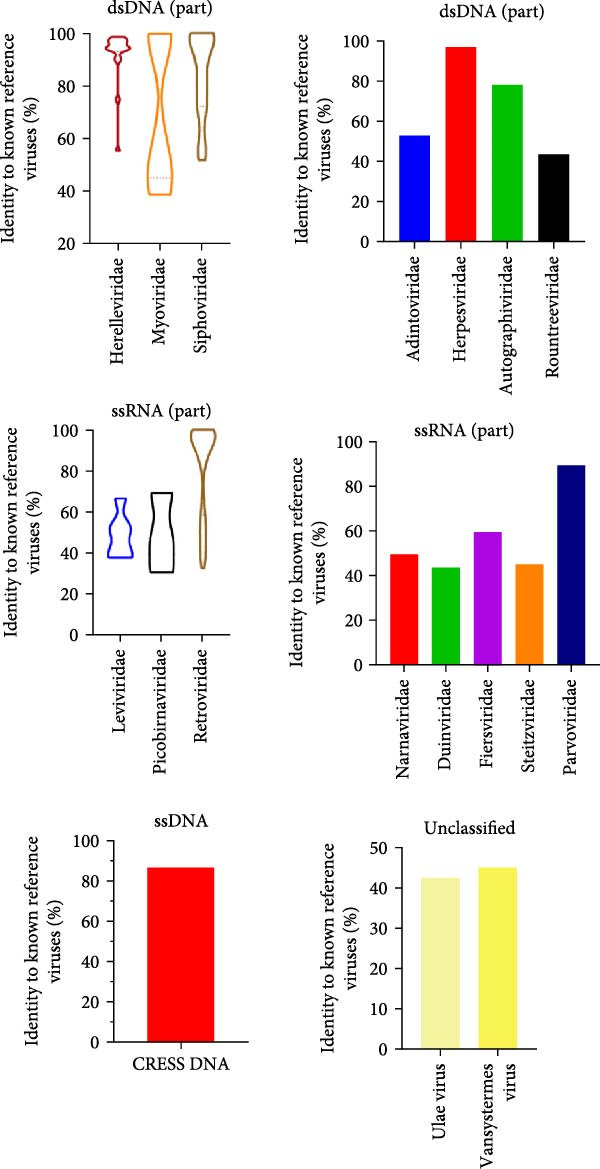
Identities of viral families from stillborn pigs with reproductive disorders compared with known reference viruses.

An outbreak of reproductive disorders occurred on a large‐scale pig farm in the Guangdong Province, which housed 3000 sows. The cause of abortion and stillbirth in these cases remained undetermined. To investigate this, tissue samples (heart and lung, etc.) were collected from stillborn fetuses. RNA was extracted from these samples and subjected to viral metagenomic sequencing analysis. The accession number for the metagenomic data is CRA024116. Sequence characteristics of the different viral communities and families of viruses detected in the samples are shown in red in the figure for the single‐stranded circular DNA viruses detected in this paper (Figure 1). Subsequently, blast protein comparisons were made between the macro viruses and the known reference viruses. The red color indicates that the detected single‐stranded cyclic DNA viruses have up to 85% similarity with a CRESS DNA fragment from NCBI, which has a GenBank number of KU043433.1 (Figure 2). Although the sequence was matched, there is no more information about this sequence in the NCBI database.

### 3.2. Sequencing of PMCV

**Figure 3:**
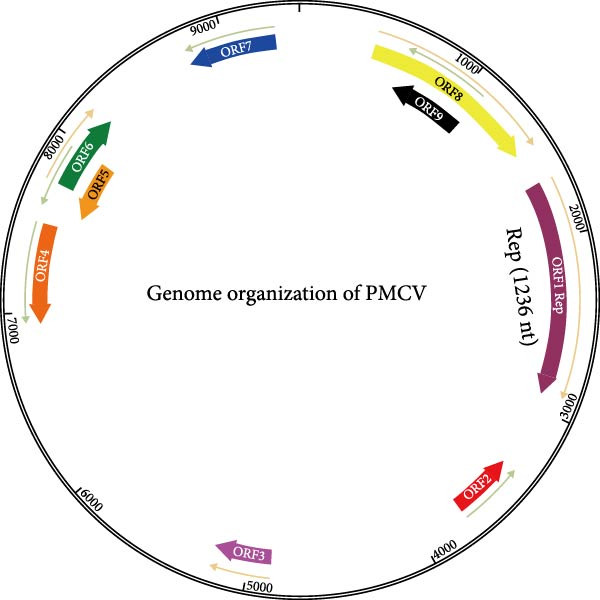
Genome organization of the porcine megalocircovirus (PMCV). PMCV has a single strand circle genome (9426 nt): the Rep (1236 nt).

Due to the short length (~400 nt) of the metagenomic fragment, we conducted PCR to directly confirm its presence in the stillborn fetuses. Subsequently, the four primer pairs were validated to be specific, efficient, and accurate for sequencing the novel circular virus. Using these four pairs of primers, we assembled complete genome of the novel circular virus (Table 2). We designed PCR primers based on this short sequence and finally amplified the whole gene of the virus with the reverse primer. We were surprised to find that the genome of the virus was 9426 nt when we put the sequence together with Snapgene7.1.2 software, which had not been reported in any other CRESS DNA viruses with such a large circular genome (Figure 3). The virus, designated PMCV, encodes 9 ORFs, and only ORF1 was identified as Rep. The complete genome sequence of the virus predicted in this study has been deposited in GenBank under accession numbers PV487790 and PV487791. The length of ORF1 of PMCV is 1236 nt, and it encodes replicase‐associated protein. We were unable to find the open reading frame of PMCV that is similar to Cap by using sequence comparison, structural modeling, and nuclear localization signal sequence searching, and so on. Due to the high GC content of the PMCV ORF7 fragment, obtaining its accurate sequence was challenging. Despite employing multiple PCR and sequencing strategies, we cannot rule out the possibility of errors in some local regions. And we speculate that the Cap of PMCV is probably distributed in this sequence. The identification of the Cap of PMCV will be important for the study of PMCV in the future.

**Table 2:** PMCV primers for sequencing.

Primer name	Primer sequence (5′ → 3′)	Size in bp
A‐F	AACATGAAAACATGGGCTAGCT	2800
A‐R	CCGGGATATTTCGGAAAGGTTAAAT	
B‐F	GACGCCATTGAAGCAAATTACTAAT	3000
B‐R	AGGTTTAGAGGCGTTCGGTTTA	
C‐F	TCCAGCACTAACAGGAACTGT	3000
C‐R	CCTGTACGGTTGCACCTC	
D‐F	TGTTTTACAAGGCGAAGGAATTCGA	2500
D‐R	CGGAATACTGTGACTGCTAGGTA	

### 3.3. Biochemical Characteristics of PMCV Rep

Structural prediction of the PMCV Rep protein revealed that it possesses the three classical domains: an N‐terminal endonuclease domain, an intermediate oligomerization domain, and a C‐terminal SF3 helicase/ATPase domain (Figure 4A). These domains exhibit a high degree of structural similarity and an identical folding pattern with PCV2 Rep (Figure 4A). The full‐length Rep could not be solubilized (Supporting Information Figure S2), which may be due to the fact that the structural domains at the N‐terminal and C‐terminal ends of the full‐length Rep would be unstable when swung in solution. Subsequent attachment of the C‐terminal domain to the oligomeric region to the GST solubilizing tag was found to be effective in promoting soluble expression, in order to verify that the C‐terminal protein of the PCMV Rep purified in this manner has good physiological properties. In order to verify the physiological properties of the PCMV Rep C‐terminal protein purified in this way, the ATPase activity of the PCMV Rep C‐terminal protein was tested by UV spectrophotometry, and the kinetic parameters of the enzyme were determined, which were fitted to the Mie equation and obtained as Vmax = 1.958 ± 0.254 nmol/min and Km = 453.6 ± 121.4 µmol/L. Based on the above results, the ATPase activity of the PCMV Rep C‐terminal protein purified from the PCMV Rep was verified. Rep C‐terminal protein has ATPase activity (Figure 4B, 4C). Size‐exclusion chromatography indicated an oligomeric state consistent with a hexamer, based on the elution volume of the major peak, suggesting that the PMCV Rep protein functions primarily in oligomeric form (data not shown). Subsequently, we predicted a hexameric model of PMCV Rep protein with its N‐terminal domain removed. In the model, each monomer is represented by a distinct color. The predicted hexamer demonstrates high structural reliability and is consistent with the previously published Rep hexamer of PCV2 (Figure 4D). These biochemical results show that the PMCV Rep protein shares significant similarity with the classical Rep protein of CRESS DNA viruses, suggesting that PMCV should be classified as a member of this viral group.

Figure 4Determination of soluble expression and ATPase activity of PMCV Rep. (a) The comparison of the predicted PMCV Rep and PCV2 Rep structure. In the predicted PMCV Rep structure, the colors blue, cyan, yellow, and orange mean very high, confident, low, and very low predicted local distance difference test (pLDDT)‐score, respectively. The pLDDT (between 0 and 100) means the distribution of per‐residue confidence score. N indicates the N terminal of the protein, and C indicates the C terminal of the protein. (b) Determination of soluble expression and ATPase activity at the C end of PMCV Rep. The expression of the oligomerization region and C‐terminal of PMCV Rep on the PGEX‐GST plasmid, and M is the molecular weight Marker; Lane 1 was 300 mM imidazole elution; Lane 2 is 50 mM imidazole; Lane 3 is 30 mM imidazole; Lane 4 is the supernatant sample through the nickel column flow sample; Lane 5 was induced precipitation; Lane 6 was the pre‐induction sample. (c) The obtained soluble target protein detected ATPase activity. (d) A hexameric model of PMCV Rep protein with its N‐terminal domain removed. In the model, each monomer is represented by a distinct color. The interface predicted template modeling score (ipTM) of the Rep model is 0.71, and the predicted Template Modeling score (pTM)) of the Rep model is 0.74.

**Figure:**
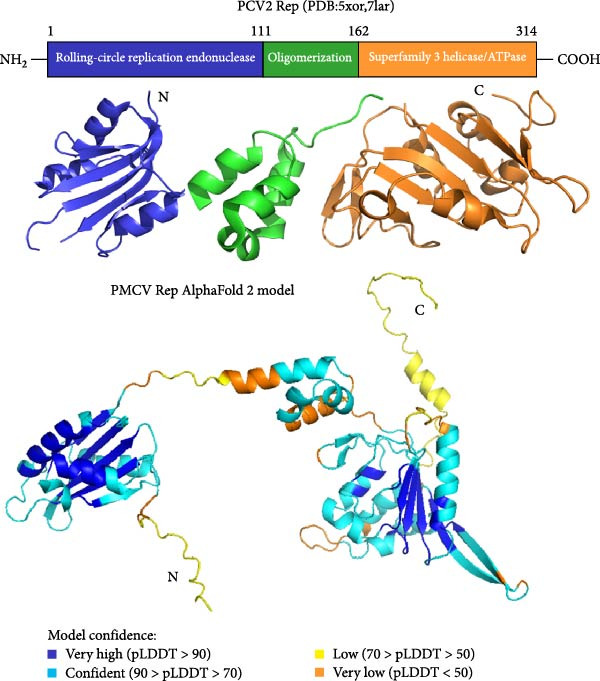
(a)

**Figure:**
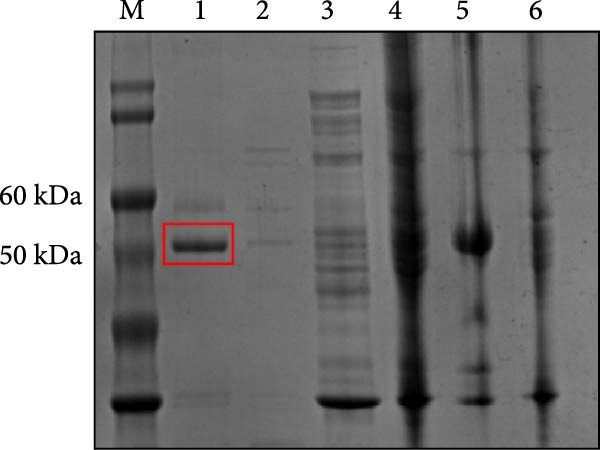
(b)

**Figure:**
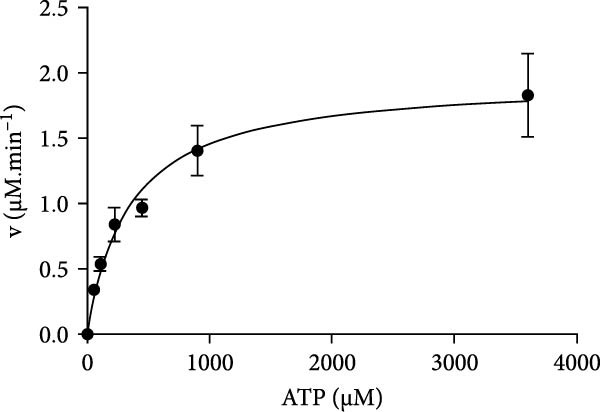
(c)

**Figure:**
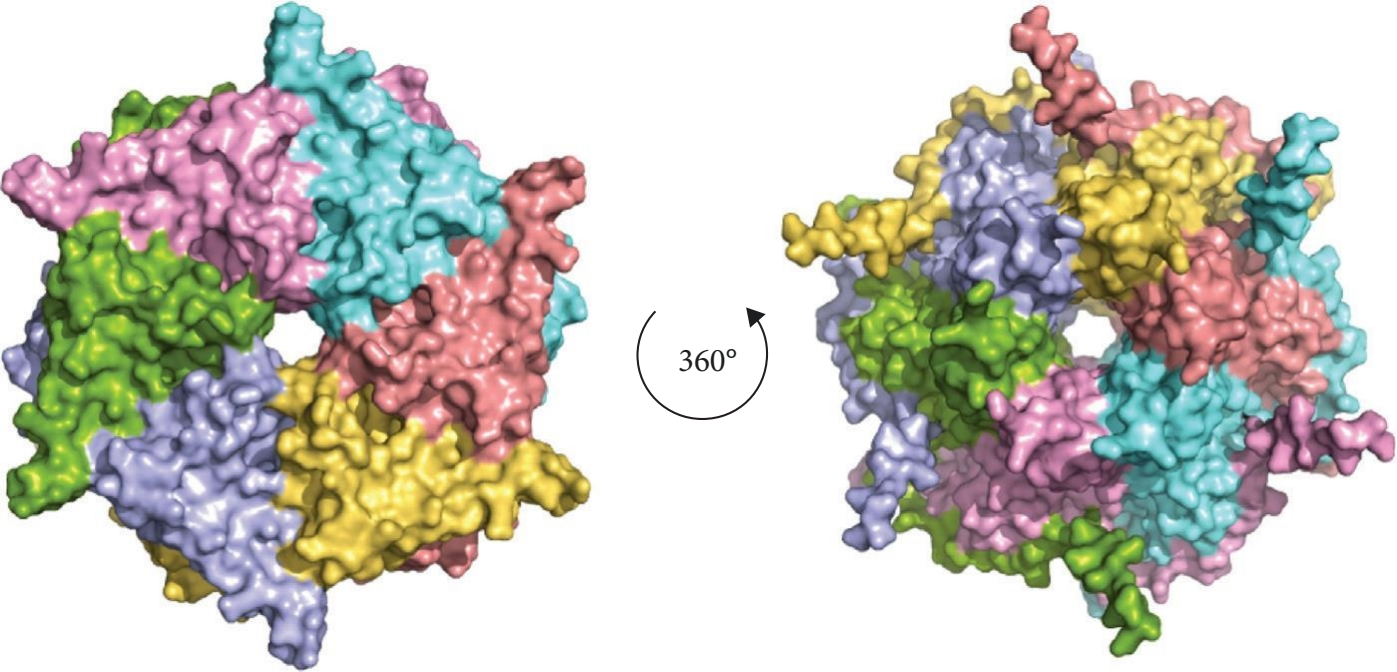
(d)

### 3.4. Phylogenetic Analysis of the Rep Amino Acid Sequences in PMCV

**Figure 5:**
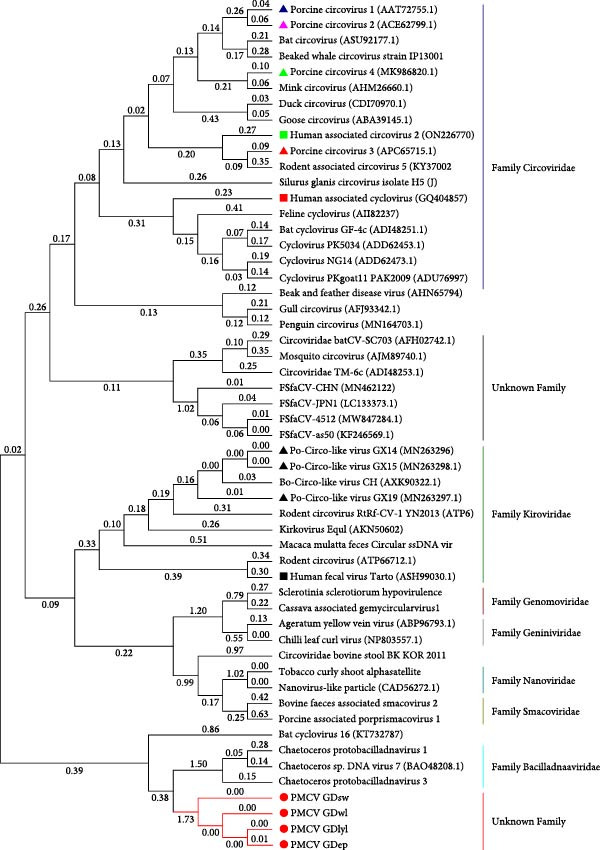
Phylogenetic tree was constructed based on Rep of CRESS DNA viruses, the green box represents the PMCV strains, and the black star indicates PCV1, PCV2, PCV3, and PCV4.

In order to genomically classify PMCV, we analyzed and investigated its conserved Rep. It was found that the nucleotide and amino acid sequences of the virus had low similarity to those of other CRESS DNA virus members. Based on the conserved Rep protein, it can be categorized into the CRESS DNA virus group. In addition, we constructed a phylogenetic tree. PMCV is genetically distant from PCV1, PCV2, PCV3, PCV4, PCLV, and several human CRESS DNA viruses and does not belong to the known family of CRESS DNA viruses (Figure 5). Furthermore, PMCV has a distinct genomic length compared to PCV1‐4, PCLV, and several human CRESS DNA viruses. The results indicate PMCV is a novel virus with largest genome in all the CRESS DNA viruses.

### 3.5. Sequence Similarity of the PMCV Rep Protein to Other CRESS DNA Viruses

**Figure 6:**
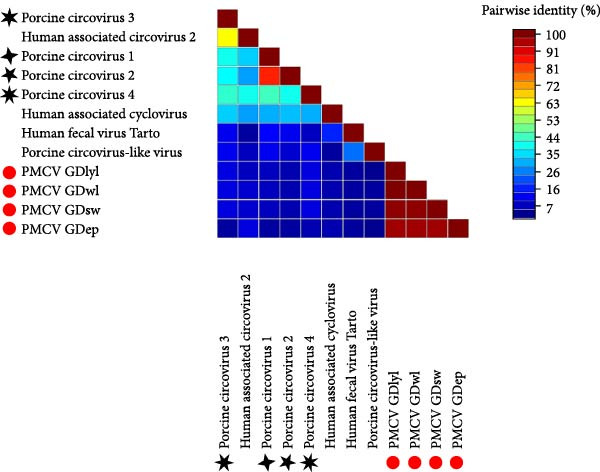
Pairwise identities of the Rep amino acids of PMCV strains to other circoviruses, the green box represents the PMCV strains, and the black star indicates PCV1, PCV2, PCV3, and PCV4.

Rep protein differed significantly from those of PCV1, PCV2, PCV3, PCV4, and several human CRESS DNA viruses, with the highest identity of 23.6% to PCV4 Rep (Figure 6). And the amino acid sequences of the Rep proteins of the four PMCV strains showed similarities ranging from 96.6% to 99.5% (Figure 6). These results indicated that PMCV is a novel CRESS DNA virus.

### 3.6. Prevalence of PMCV in Swine Population

**Figure 7:**
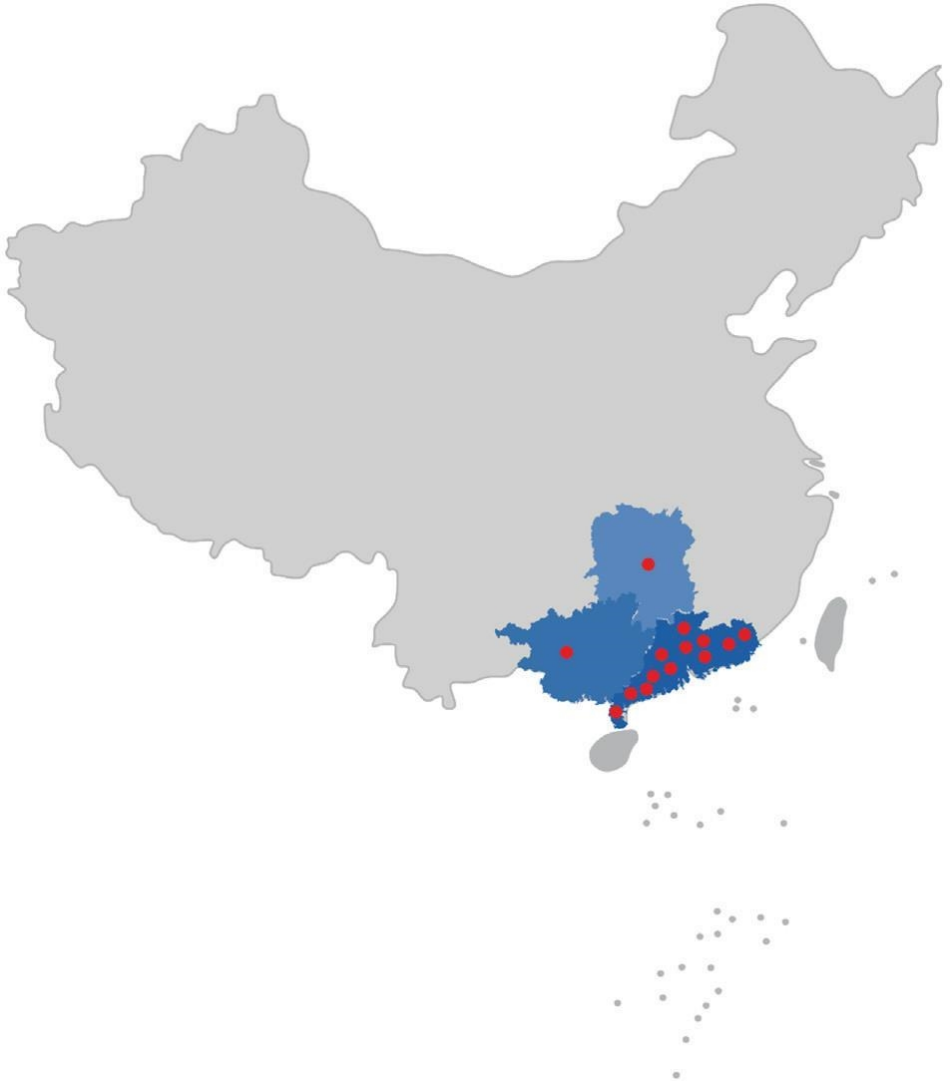
Geographic distribution of the PMCV. Areas where surveillance of PMCV was carried out are shown in blue. Red circles indicated the locations of farms with confirmed PMCV infection. A total of 14 pig farms in the Guangdong, Hunan, and Guangxi provinces were PMCV positive.

We initiated epidemiologic and pathogenicity surveillance of PMCV. A total of 125 samples were collected. PMCV was detected in 14 pig farms in different areas, and the positive farms were distributed in the Guangdong, Guangxi Province, and Hunan Province (Figure 7). Positive PMCV farms were found to be 41.18% (14/34), and positive PMCV samples were found to be 24% (30/125). Although PMCV positivity rates varied across samples, and PMCV1 was detected mainly in feces and stillbirths (Table 3), which further suggests that PMCV is closely associated with reproductive disorders and is in agreement with the viral metagenomic sequencing data. These results also suggest that PMCV associated with PCVAD has become endemic in pig herds.

**Table 3:** Statistics of samples for PMCV detection.

Sample type	Total number of samples	Number of positives	Detection rate (%)
Feces	7	4	57.14
Cotton swab (with twine)	55	22	40.00
Serum	41	2	4.88
Disease material	4	0	0.00
Aborted fetus	7	2	28.57
Tissue	11	0	0.00
Total	125	30	24

## 4. Discussion

The taxonomic landscape of eukaryotic CRESS DNA viruses has expanded significantly, with GenBank records demonstrating a two‐fold increase in identified species over the past decade [2]. This trend is exemplified by emerging porcine circoviruses (PCVs), including PCV3 and PCV4, which exhibit marked genomic divergence (<30% homology) from classical PCV1/PCV2 strains and were initially isolated during disease outbreaks in swine farms [26, 27]. The ecological or host impact of CRESS DNA viruses remains largely unknown [2, 28, 29]. CRESS DNA viruses have been found in a variety of hosts, including cattle [30], chimpanzees [31], bats [32], chickens [33], humans [34], and pigs [35]. The novel circovirus described in this study demonstrates even greater phylogenetic distance from all known Circoviridae members (PCV1‐4), challenging current classification frameworks. Rep protein differed significantly from those of PCV1, PCV2, PCV3, PCV4, and several human CRESS DNA viruses, with the highest identity of 23.6% to PCV4 Rep. Despite low amino acid sequence similarity, the replication‐associated proteins (Rep) of various CRESS DNA viruses exhibit a highly conserved structure. The identification of complete genome of PMCV highlights the expanding genetic diversity of porcine CRESS DNA viruses.

Establishing a quantitative detection method for viral nucleic acids is of great significance for new viruses. Initial attempts to develop a quantitative PCR assay targeting the PMCV Rep gene revealed unexpected cross‐reactivity with PCV2 and PCV3, suggesting conserved nucleotide motifs between these different CRESS DNA viruses (Supporting Information Figures S3, S4). Commercially available PCV2 antigen detection kits indicated a positive result for this clinical sample. However, conventional PCR failed to amplify the complete PCV2 genome. Subsequent testing for PMCV revealed that the sample was positive. Sequencing further confirmed that the obtained sequence was the Rep of PMCV. This also revealed that some of the nucleotide sequences in the Rep of PCV2 would be similar to the sequence of the Rep of PMCV, which affected the establishment of the detection method. Technical obstacles were compounded by PMCV’s genomic characteristics: (1) extreme GC heterogeneity complicating sequencing efforts, (2) limited availability of complete genomes restricting alternative primer design. These constraints currently preclude reliable antigen detection method establishment.

PMCV may infect multiple organs in pigs. PMCV was detected mainly in feces and stillborn fetuses, further suggests the close relationship between PMCV and reproductive disorders, which is consistent with viral metagenomic sequencing analysis. Focus needs to be paid to associated transmission routes. It has been suggested that viruses that are not directly pathogenic may weaken the host’s immune function and trigger infection with other pathogenic agents [36]. The overall detection rate for serum samples is low and may need to be analyzed in conjunction with the test method or sampling time. The high farm‐level prevalence and sample positivity rate of PMCV demonstrate its wide circulation in southern China. In the future, more in‐depth and comprehensive epidemiological investigations on PMCV in different regions and seasons are needed.

Structurally, PMCV encodes nine ORFs with no identifiable homologs to canonical Cap proteins through BLASTp, structural modeling, or nuclear localization signal analyses. Intriguingly, ORF7—a GC‐rich region resistant to conventional PCR amplification—may harbor atypical Cap encoding sequences. Furthermore, all attempts to isolate the virus were not successful under these experimental conditions. Consequently, the inability to isolate the virus hindered both morphological observation of virus particles and the development of an animal disease model.

In conclusion, we report a novel PCV associated with pig reproductive disorder, describing its genome‐wide characterization and preliminary epidemiological investigations. Therefore, the pathogenic role of PMCV in pig herds clearly warrants further investigation.

## 5. Conclusions

The discovery of a novel large‐genome PCV, designated PMCV, signifies a new dimension in PCV diversity, characterized by substantial phylogenetic divergence from all known members (including PCV1‐4 and several human circoviruses), which challenges existing taxonomic boundaries. Its detection in stillborn fetuses further implies a potential role in reproductive disorders, consistent with the clinical impact of other circoviruses. Resolving PMCV’s transmission dynamics, pathogenicity, and coinfections is therefore a crucial next step. These findings collectively call for a timely update of diagnostic and taxonomic systems to encompass the increasing complexity of CRESS DNA viruses in pig population.

## Ethics Statement

All experiments were approved by the China Local Ethical Committee (Approval Numbers SYXK2019‐0136 and 2022E018).

## Conflicts of Interest

The authors declare no conflicts of interest.

## Author Contributions

**Xianhui Liu**, **Lin Wang, Xinming Zhang, and Yilong Liu**: conceptualization, data curation, formal analysis. **Yixuan Li, Zan li, and Jian Xiao**: nvestigation, methodology, project administration.**Yanling Liu, Pengshuai Liang, and Leyi Zhang**: software, supervision, validation, visualization. **Zheng Xu, Yebing Liu, and Changxu Song**: funding acquisition, resources, writing – original draft, writing – review and editing. All authors contributed to experimental design. **Xianhui Liu**, **Yixuan Li, and Jian Xiao** contributed equally to this work and are co‐first authors.

## Funding

This work was supported by the Guangdong Provincial Agricultural Breeding Technique Innovation Project (2024‐XBH‐00‐003).

## Supporting Information

Additional supporting information can be found online in the Supporting Information section.

## Supporting information


**Supporting Information** Supporting Information Figure 1. Clinical presentation of PMCV infection in stillborn samples. Clinically affected sows demonstrated with reproductive failure by stillborn. Supporting Information Figure 2. Electrophoresis results of the PMCV Rep full‐length protein, M is molecular weight Marker; Lane 1 was 300 mM imidazole elution; Lane 2 is 50 mM imidazole; Lane 3 is 30 mM imidazole; Lane 4 is the supernatant sample through the nickel column flow sample; Lane 5 was induced precipitation; Lane 6 was the preinduction sample. Supporting Information Figure S3. The nucleotide sequence comparison between the PCV2 Rep and the PMCV Rep. Supporting Information Figure S4. The nucleotide sequence comparison between the PCV3 Rep and the PMCV Rep.

## Data Availability

The genome sequences identified in this study have been deposited in the GenBank database (PV487790 and PV487791).
