# Supplementary material for: Emergence of a Novel CRESS‐DNA Virus Associated with Swine Reproductive Failure in China
Source: Transbound Emerg Dis. 2026 Feb 6;2026:4053892. doi: 10.1155/tbed/4053892 (PMC12881700; doi:10.1155/tbed/4053892)
Supplement: Supplementary file 1 — Supporting Information Supporting Information Figure 1. Clinical presentation of PMCV infection in stillborn samples. Clinically affected sows demonstrated with reproductive failure by stillborn. Supporting Information Figure 2. Electrophoresis results of the PMCV Rep full‐length protein, M is molecular weight Marker; Lane 1 was 300 mM imidazole elution; Lane 2 is 50 mM imidazole; Lane 3 is 30 mM imidazole; Lane 4 is the supernatant sample through the nickel column flow sample; Lane 5 was induced precipitation; Lane 6 was the preinduction sample. Supporting Information Figure S3. The nucleotide sequence comparison between the PCV2 Rep and the PMCV Rep. Supporting Information Figure S4. The nucleotide sequence comparison between the PCV3 Rep and the PMCV Rep. [file TBED-2026-4053892-s001.docx]

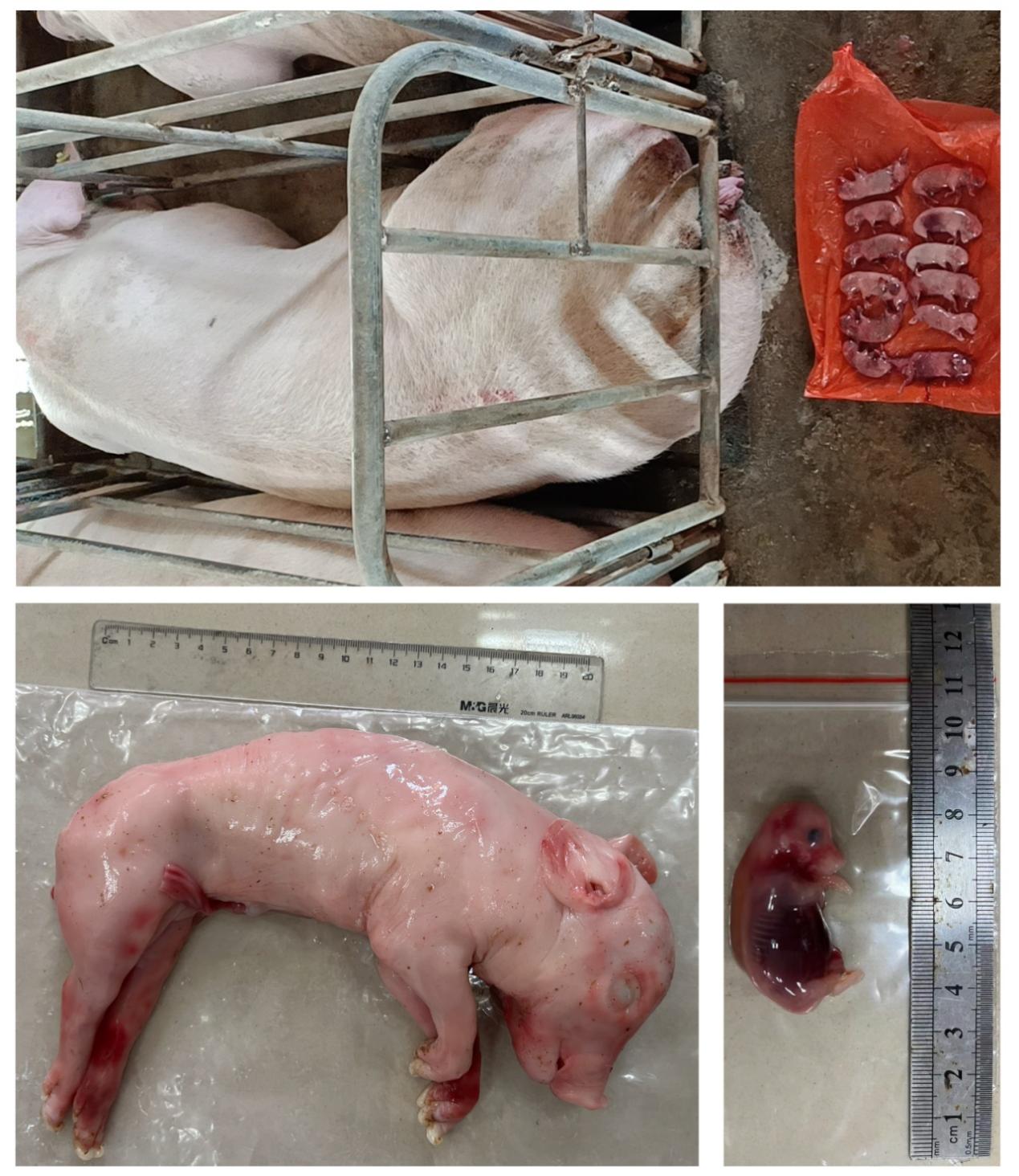


**Supplementary Fig 1.** **Clinical presentation of PMCV infection in stillborn samples.** Clinically affected sows demonstrated with reproductive failure by stillborn.


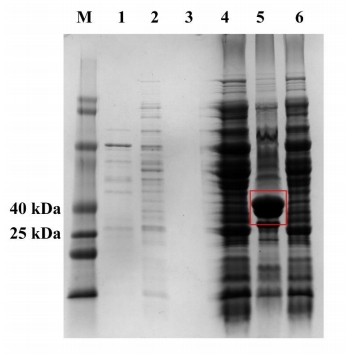


**Supplementary Fig 2.** Electrophoresis results of the PMCV Rep full-length protein, M is molecular weight Marker; Lane 1 was 300 mM imidazole elution; Lane 2 is 50 mM imidazole; Lane 3 is 30 mM imidazole; Lane 4 is the supernatant sample through the nickel column flow sample; Lane 5 was induced precipitation; Lane 6 was the pre-induction sample.


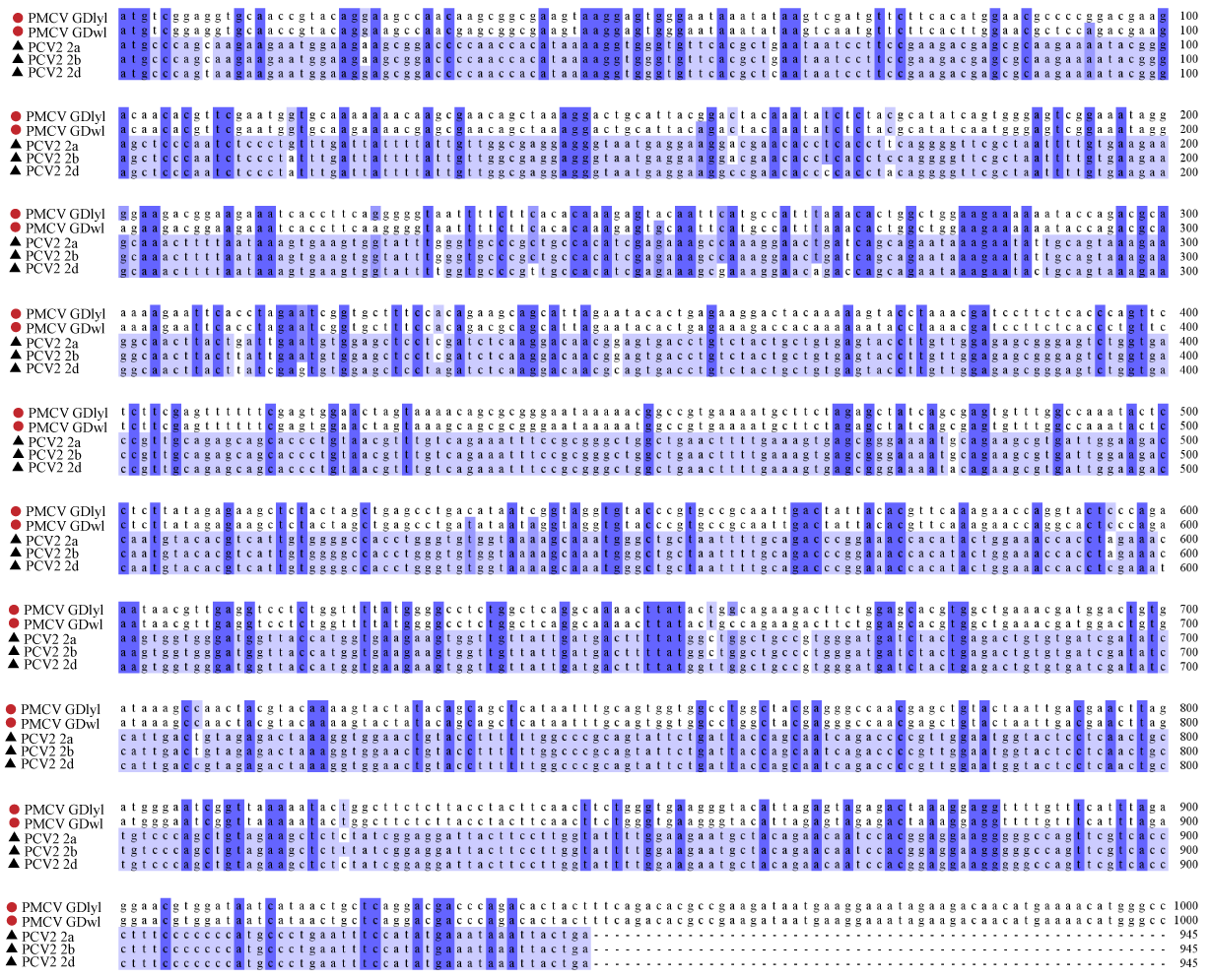


**Supplementary Fig S3.** The nucleotide sequence comparison between the PCV2 Rep and the PMCV Rep.


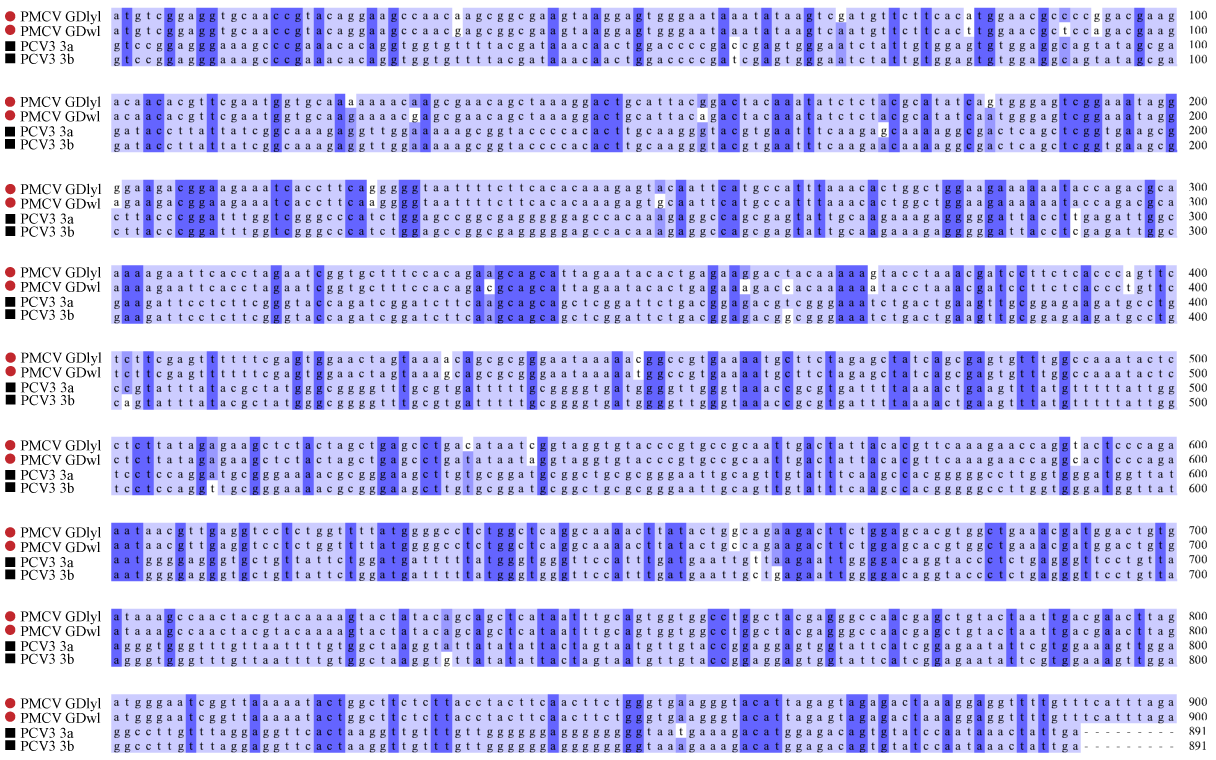


**Supplementary Fig S4.** The nucleotide sequence comparison between the PCV3 Rep and the PMCV Rep.
